# Geometry Optimization of Thermoelectric Modules: Deviation of Optimum Power Output and Conversion Efficiency

**DOI:** 10.3390/e22111233

**Published:** 2020-10-29

**Authors:** Mario Wolf, Alexey Rybakov, Richard Hinterding, Armin Feldhoff

**Affiliations:** Institute of Physical Chemistry and Electrochemistry, Leibniz University Hannover, Callinstraße 3A, D-30167 Hannover, Germany; alexey.rybakov@pci.uni-hannover.de (A.R.); richard.hinterding@pci.uni-hannover.de (R.H.)

**Keywords:** thermoelectric materials, energy harvesting, thermoelectric generator, working points, maximum electrical power point

## Abstract

Besides the material research in the field of thermoelectrics, the way from a material to a functional thermoelectric (TE) module comes alongside additional challenges. Thus, comprehension and optimization of the properties and the design of a TE module are important tasks. In this work, different geometry optimization strategies to reach maximum power output or maximum conversion efficiency are applied and the resulting performances of various modules and respective materials are analyzed. A Bi_2_Te_3_-based module, a half-Heusler-based module, and an oxide-based module are characterized via FEM simulations. By this, a deviation of optimum power output and optimum conversion efficiency in dependence of the diversity of thermoelectric materials is found. Additionally, for all modules, the respective fluxes of entropy and charge as well as the corresponding fluxes of thermal and electrical energy within the thermolegs are shown. The full understanding and enhancement of the performance of a TE module may be further improved.

## 1. Introduction

The direct energy conversion from wasted thermal energy into usable electrical energy via thermoelectric (TE) modules has been extensively studied and improved in recent years. Such devices benefit from long-term stability without the need of maintenance and they are quietly operating without moving parts that may get damaged over time [1]. The main parts of research on thermoelectric energy conversion are investigating and improving thermoelectric materials in order to reach high power output and high conversion efficiency on the one hand [2,3] and the scalable and effective manufacturing of devices on the other hand [4,5]. However, up to now, TE modules have not achieved characteristics that justify the investment for a wide commercial usage. Especially, the design of the device, the optimization of the cross-sectional area ratio, and thermal and electrical contact resistivity are crucial factors on the way from a promising material to a functional device with high power output and conversion efficiency [6], even if suitable thermoelectric materials are provided. The aim of the work is to improve the understanding and the optimization of the working principle of TE modules based on finite element method (FEM) simulations of several material combinations with the software ANSYS for various geometry optimization strategies.

### 1.1. Thermoelectric Materials

The thermoelectric energy conversion can be described by the coupling of the flux density of electric charge ***j***_q_ and the flux density of entropy ***j***_s_. These fluxes are transmitted by the thermoelectric material tensor, which represents the characteristics of the included thermoelectric materials with a cross-sectional area *A* and length *l*, when simultaneously placed in a gradient of electrical potential ∇φ and a gradient of temperature ∇*T*, as shown in Equation (1) [7,8].

(1) $$\left(\begin{array}{c} j_{q}\\ j_{s} \end{array}\right)=\frac{A}{l}\cdot \left(\begin{array}{cc} \sigma & \sigma \cdot \alpha \\ \sigma \cdot \alpha & \sigma \cdot \alpha^{2}+\Lambda_{OC} \end{array}\right)\cdot \left(\begin{array}{c} -\nabla \varphi \\ -\nabla T \end{array}\right)$$

The energy conversion is therefore mainly based on three material parameters: the isothermal electrical conductivity σ, the Seebeck coefficient α and the entropy conductivity at electrical open-circuit Λ_OC_. In principle, all three quantities are tensors themselves, but, for homogeneous materials, they are often treated as scalars [8,9]. The figure of merit *f* = *zT* [10,11] shown in Equation (2), which displays the conversion efficiency of a thermoelectric material, is a function of the three material parameters.

(2) $$f=\frac{\sigma \cdot \alpha^{2}}{\Lambda_{OC}}=\frac{\sigma \cdot \alpha^{2}}{\lambda_{OC}}\cdot T=zT$$

Consequently, thermoelectric materials are usually desired to have a high power factor σα^2^ and a simultaneously low open-circuited entropy conductivity Λ_OC_. Note that, due to the use of entropy conductivity Λ_OC_ instead of the heat conductivity λ_OC_, the absolute temperature *T* does not occur explicitly within the short form of Equation (2), but implicitly within the three material parameter σ(*T*), α(*T*) and Λ_OC_(*T*) [11].

**Figure A1:**
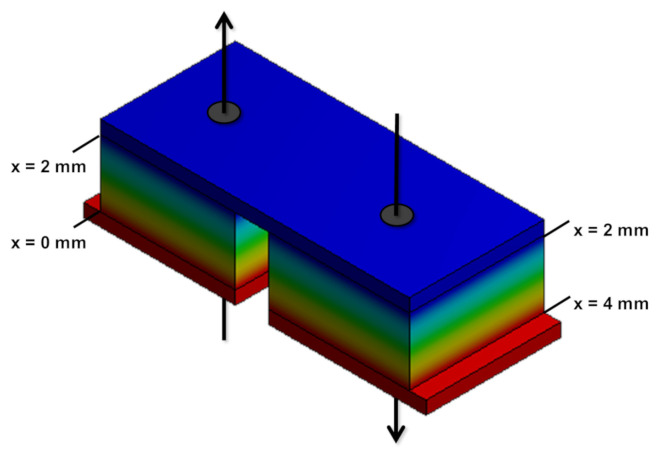
Analyzed path *x* along a central line through the respective thermoleg on the example of module 1.

Within the thermoelectric materials, the respective flux density of thermal energy *j*_E,th_(*x*) and flux density of electrical energy *j*_E,el_(*x*) at a certain point x across the length of the materials are given as the product of the respective flux density of entropy *j*_S_(*x*) and flux density of electrical charge *j*_q_(*x*) and the temperature *T*(*x*) and voltage *U*(*x*) = Δφ(*x*) at this point (Equations (3) and (4)) [8]. Note that this description is analyzed as a function of *x*, along a central line through the respective thermoleg (compare Figure A1), so these values as a function of *x* are used as scalars.

(3) $$j_{E,\mathrm{th}}\left(x\right)=j_{S}\left(x\right)\cdot T\left(x\right)$$

(4) $$j_{E,\mathrm{el}}\left(x\right)=j_{q}\left(x\right)\cdot U\left(x\right)$$

These descriptions of electrical and thermal phenomena are used as a basis to analyze and improve the understanding of thermoelectric modules within this work. Here, the explicit description of the flux densities of charge and entropy and the resulting flux densities of thermal and electrical energy can be useful in order to further understand and improve the thermoelectric energy conversion.

As thermoelectric materials, various classes of materials have been studied intensively including bismuth telluride [12,13], which is commonly used for thermoelectric modules, other tellurides [14], and selenides [15,16], intermetallic phases, such as as Zintl phases [17,18,19] and half-Heusler phases [20,21], oxides and oxyselenides [22,23], and conductive polymers [24,25]. Each material class provides a different thermoelectric characteristic, requires special treatments or fabrication and it is suitable in a certain application temperature range [2]. In order to influence and improve the thermoelectric properties, band structure modelling via doping and nanostructuring [26,27,28], segmentation of thermoelegs [29,30,31] and the utilization of hybrid materials [32,33,34] are widely investigated.

**Figure 1:**
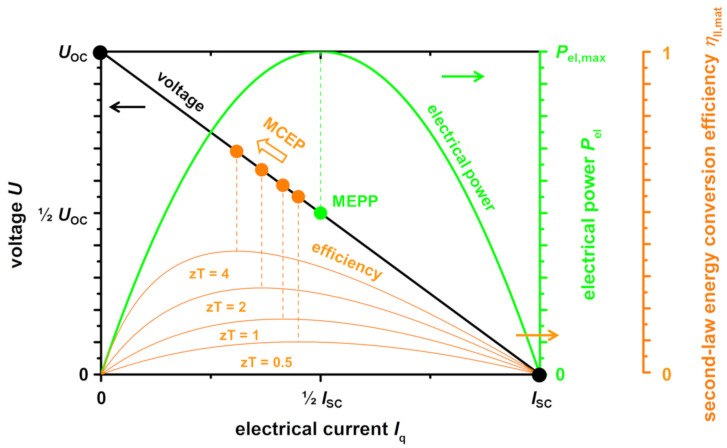
Normalized *U*-*I*_q_ and *P*_el_-*I*_q_ characteristics of some hypothetic thermoelectric materials with a *zT* of 0.5, 1, 2, and 4. The second-law energy conversion efficiency η_II,mat_ increases with increasing figure of merit *zT*. The maximum conversion efficiency point (MCEP) is a function of the figure of merit *zT* and, therefore, drifts apart from the maximum electrical power point (MEPP). Working points of short-circuit (SC) with the short-circuit current *I*_q,SC_ and open-circuit (OC) with the open-circuit voltage *U*_OC_ are marked. Reworked from [11].

The resulting thermoelectric performance of a material is usually described by the *U*-*I*_q_-characteristic (voltage-electrical current curve) and the resulting electrical power curve *P*_el_-*I*_q_. Here, two important material working points can be identified: the maximum electrical power point (MEPP) of a respective material (the point where *P*_el_ = *U* ·*I*_q_ reaches its maximum), which is given at half the open-circuited voltage *U*_OC_, and half the short-circuited current *I*_q,SC_ and the maximum conversion efficiency point (MCEP) of a respective material, which is a function of the figure of merit *zT* of the material. The MCEP and MEPP drift apart with increasing figure of merit *zT* of the respective material, As shown in a previous work [11] (Figure 1). Therefore, optimizing different parameters to influence the materials MEPP and MCEP are important to effectively improve the performance of a resulting TE module. Furthermore, this implies that not only the resulting conversion efficiency based on the figure of merit *zT*, but also the resulting electrical power output, which is a function of the power factor σα^2^, is a key parameter. In fact, the power factor should have at least the same significance as the figure of merit *zT*, as has been reported before [2,35].

### 1.2. From Material to Device

In this work, the concept of the material working points MEPP and MCEP and the resulting significance of figure of merit *zT* and power factor σα^2^ are transferred to a TE module. As described before, the concept and design of a TE module also strongly influence the resulting performance. This is based on several factors:

- The respective thermoelectric materials properties.
- The design of the respective device, the flexibility and the free volume.
- The aimed application temperature range, limiting the options for thermoelectric materials.
- Optimization factors, such as thermal- and electrical-contact resistivity, as well as the cross-sectional area ratio between *n*- and *p*-type materials

Especially, the respective geometry of the *p*- and *n*-type materials strongly influence the certain MEPP and MCEPs of the materials and therefore the resulting performance of the TE module [6]. Often, the geometry is optimized to a maximum figure of merit *zT* and the resulting *A*_n_/*A*_p_ ratio is used for simulations for example by Ouyang and Li [30]. For certain materials, this optimization, in fact, leads to overlapping MCEP and MEPPs of the respective materials in a resulting module due to matching values of the thermal conductivity λ_n_ = λ_p_ [36], which, however, is not always the case. Recently, Xing et al. [36] also described that an optimization of TE modules for a high power output and an according materials choice can strongly enhance the resulting properties when compared to an optimization for maximum energy conversion efficiency. This corresponds to the assertion of the significance of the power factor. Therefore, in this work, an analysis of different material combinations in a TE module is provided, based on the analogous description of *j*_E,th_(*x*) and *j*_E,el_(*x*) shown above for three different optimization strategies: for maximum *zT*, for matching *I*_q,SC_ (and, therefore, overlapping material working points), and for maximum electrical power output. For this purpose, FEM simulations of various modules are provided both based on materials with similar (Bi_2_Te_3_-based TE module and half-Heusler-based TE module), as well as with very different thermoelectric properties (oxide-based TE module) of the *n*- and *p*-type materials.

## 2. Methods and Simulation

### 2.1. Materials and Modules for FEM Simulations

**Figure 2:**
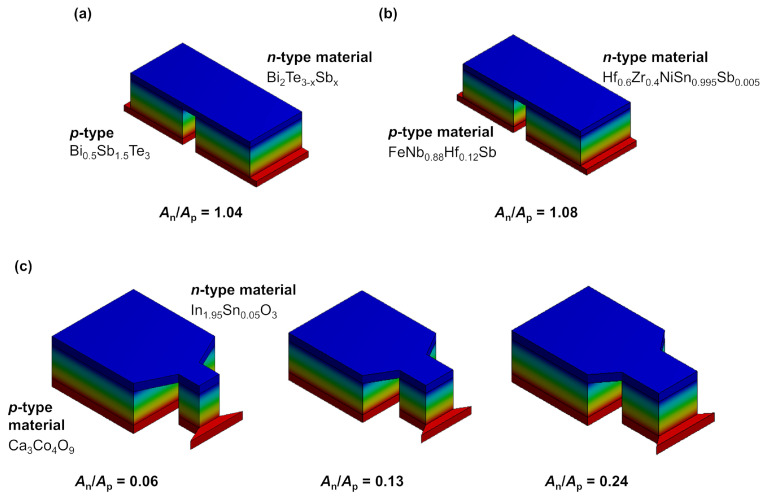
Resulting modules characterized via finite elemente simulations (FEM)-simulations. (**a**) Bi_2_Te_3_-based TE module 1, (**b**) half-Heusler-based TE module 2, and (**c**) oxide-based TE module 3 with three different *A*_n_/*A*_p_ ratios. The colors refer to the respective temperatures (red: hot side, blue: cold side). Note that the effective area *A*_n_ + *A*_p_ is constant for all modules and *A*_n_/*A*_p_ ratios. As connector, the characteristics of copper has been used in the simulation.

Table 1 shows the used materials. For all thermoelectric materials, literature data have been used. The exact input values are shown in Table A1, Table A2 and Table A3 in Appendix A. As a connector, a metal conductor made of copper with 0.5 mm height, an electrical conductivity of 4.85 × 10^8^ S m^−1^ and a thermal conductivity of 400 W m^−1^ K^−1^ was used. Figure 2 shows the resulting TE modules used for FEM simulations.

### 2.2. Optimization of Geometry

The *A*_n_/*A*_p_ ratios for the simulated modules have been calculated for three different optimizations: First, according to a *zT* optimization for maximum energy conversion efficiency that has been derived and used before (Equation (5)) [30]. Here, ρ_n_ and ρ_p_ are the specific electrical resistivity and λ_n_ and λ_p_ the heat conductivity of the *n*- and *p*-type materials, respectively:

(5) $${\left[\frac{A_{n}}{A_{p}}\right]}_{\mathrm{zT}}=\sqrt{\frac{\rho_{n}}{\rho_{p}}\cdot \frac{\lambda_{p}}{\lambda_{n}}}$$

Second, the $\left[\frac{An}{Ap}\right]$_matching *I*_q,SC__ ratio for overlapping material working points was calculated according to Equation (6) (compare Equations (A1)–(A7) in Appendix B). Here, α_n_ and α_p_ are the Seebeck coefficient of the *n*-type and *p*-type materials, respectively:

(6) $${\left[\frac{A_{n}}{A_{p}}\right]}_{\mathrm{matching}I_{q,SC}}=\frac{\alpha_{p}}{|\alpha_{n}|}\cdot \frac{\rho_{n}}{\rho_{p}}$$

Third, an optimization for maximum power output was conducted according to Xing et al. [36] via Equation (7) (compare Equations (A8)–(A14) in Appendix C):

(7) $${\left[\frac{A_{n}}{A_{p}}\right]}_{power}=\sqrt{\frac{\rho_{n}}{\rho_{p}}}$$

Additionally, the areas of the *n*- and *p*-type materials have been chosen for the same effective area *A*_n_ + *A*_p_ for all modules. The maximum first-law energy conversion efficiency η_I,TEG,max_ for all optimized geometries have been calculated from the thermoelectric properties of the materials [9,11,30] (compare Equations (A15)–(A19) in Appendix D). The length of all thermolegs was chosen to be *l* = 2 mm, as otherwise there would have been too many varying parameters and a fixed and matching length for *n*- and *p*-type is reasonable for a functional TE module.

### 2.3. Simulation Parameters

The software ANSYS Mechanical (Version 2020 R1), which is based on the finite element method, is used in order to simulate the TE modules. Here, a steady-state thermal-electrical conduction analysis that allows for a simultaneous solution of thermal and electrical fields was chosen. After setting the material parameters for the *n*- and *p*-type thermolegs, the following boundary conditions for the simulation were set: the temperature of the cold junction, the ambient temperature that is equal to the temperature of the cold junction, the side at zero potential, and the side that determines the value of the electric current; all of the remaining faces were set for free convection in air with the heat transfer coefficient with a typical value of 20 W m^−2^ K^−1^ [42].

**Figure A2:**
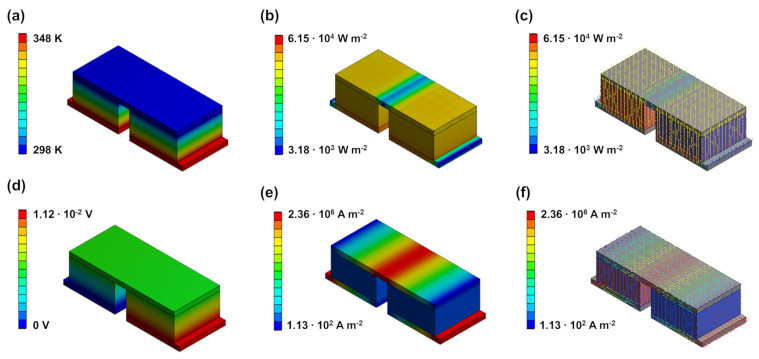
Distribution of (**a**) temperature *T*(*x*), (**b**,**c**) flux density of thermal energy *j*_E,th_(*x*), (**d**) voltage *U*(*x*) and (**e**,**f**) flux density of electrical charge *j*_q_(*x*) in module 1.

**Figure A3:**
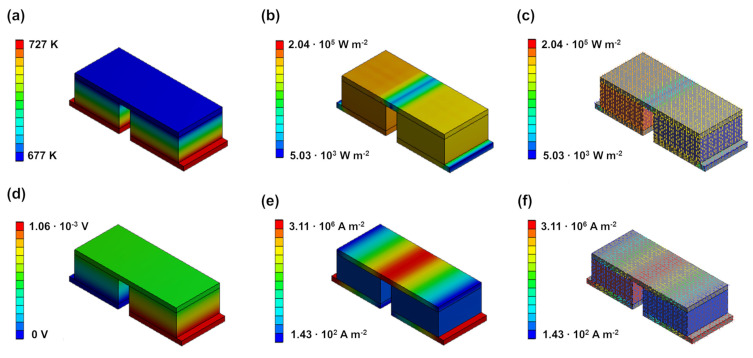
Distribution of (**a**) temperature *T*(*x*), (**b**,**c**) flux density of thermal energy *j*_E,th_(*x*), (**d**) voltage *U*(*x*) and (**e**,**f**) flux density of electrical charge *j*_q_(*x*) in module 2.

**Figure A4:**
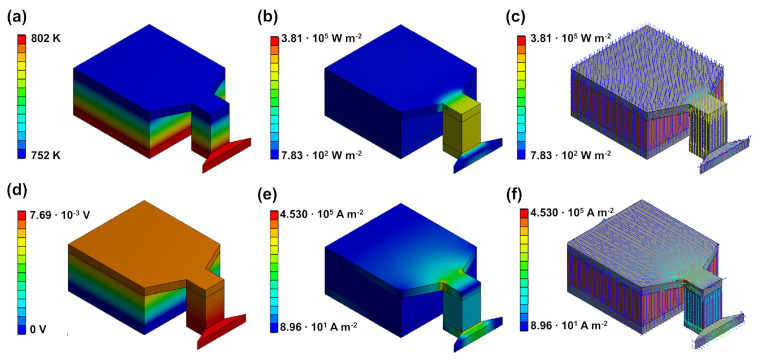
Distribution of (**a**) temperature *T*(*x*), (**b**,**c**) flux density of thermal energy *j*_E,th_(*x*), (**d**) voltage *U*(*x*) and (**e**,**f**) flux density of electrical charge *j*_q_(*x*) in module 3 with *zT*-optimized geometry.

**Figure A5:**
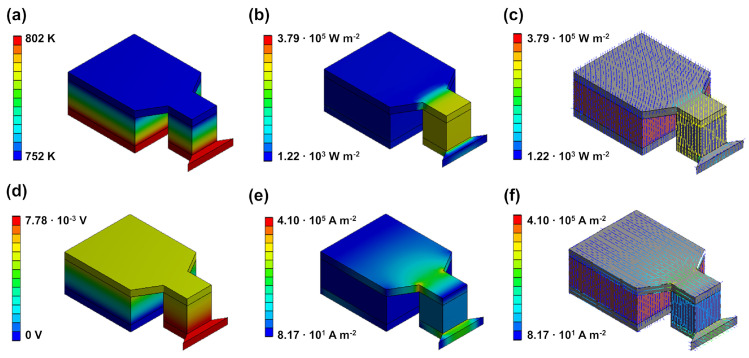
Distribution of (**a**) temperature *T*(*x*), (**b**,**c**) flux density of thermal energy *j*_E,th_(*x*), (**d**) voltage *U*(*x*) and (**e**,**f**) flux density of electrical charge *j*_q_(*x*) in module 3 with geometry for matching *I*_q,SC_.

**Figure A6:**
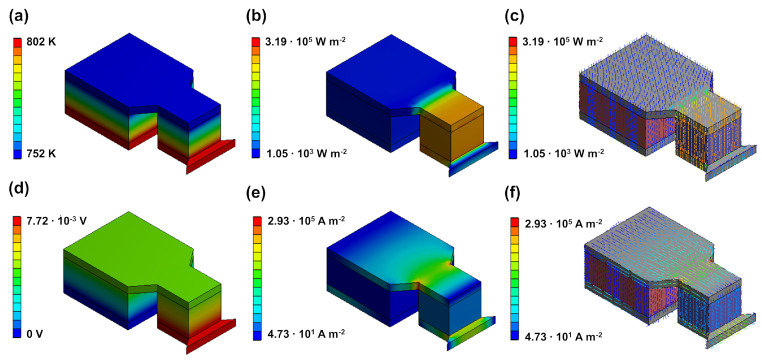
Distribution of (**a**) temperature *T*(*x*), (**b**,**c**) flux density of thermal energy *j*_E,th_(*x*), (**d**) voltage *U*(*x*) and (**e**,**f**) flux density of electrical charge *j*_q_(*x*) in module 3 with power-optimized geometry.

The simulation process was divided into two stages. First, a *U*-*I*_q_ curve was taken in order to evaluate the general characteristics of the TE module. By changing the value of the electrical current that can flow through the TE module, the effect of the external load on the voltage is simulated. Using the *U*-*I*_q_ curve, the electrical power *P*_el_ was calculated and a *P*_el_-*I*_q_ curve was constructed to determine the MEPP. Then, to study the specific characteristics of the TE module at the MEPP, the following four distributions were simulated: temperature, flux density of thermal energy, electrical voltage, and flux density of charge. From each distribution, the values alongside the center of the thermoleg have been calculated. For these positions inside the leg, the local entropy flux density was calculated from the local temperature and local flux density of thermal energy according to Equation (3). The values of the electrical voltage and the local flux density of electrical charge were used in order to calculate the flux density of the electrical energy according to Equation (4). As a result, a description of all parameters as a function of the position *x*, along a central line through the respective thermoleg, is received. The corresponding images of the distribution of the temperature *T*(*x*), the voltage *U*(*x*), flux density of thermal energy *j*_E,th_(*x*), and flux density of electrical charge *j*_q_(*x*) within the thermolegs are shown in Figure A2, Figure A3, Figure A4, Figure A5 and Figure A6 in the Appendix E.

### 2.4. Notes on Limitations

For all material parameters, a linear behavior within the applied temperature range has been assumed and the average value has been used for the calculation of the *A*_n_/*A*_p_ ratio. Over a relatively small temperature difference of 50 K, the assumption of linear behavior of the thermoelectric parameters can be made, but, for exact simulations, the respective behavior has to be analyzed in detail for each specific case. Because the maximum temperature difference in the simulation was only 50 K and the maximum application temperature was about 1000 K, the dominant mechanism of heat transfer is convection, so the influence of thermal radiation was not considered. Note that, for temperatures above 1000 K and if ceramic substrates are used on top and at the bottom, the thermal radiation becomes increasingly important and has to be considered if an application at higher temperatures is aimed. For all of the simulated modules, an active cooling with a stable temperature difference of 50 K was assumed. Although a matching length *l* for both thermolegs is reasonable, this may also be optimized, since the length strongly influences the *U*–*I*_q_-curve as well as the temperature difference, if no active cooling with a stable temperature difference is applied. Additionally, as mentioned before, the electric and thermal contact resistivity between each individual thermoleg and the connector is an important parameter, which has to be investigated and optimized for each individual case. To allow for comparison, ideal contacts are assumed in this work. The results in this work are specifically shown for a thermoelectric module in generator mode, which, however, may also apply for the entropy pump mode in thermoelectric coolers. For the thermoelectric materials, the respective material working points are also correlated to the material properties [11], but, for the thermoelectric modules in entropy pump mode, this is yet to be proven.

## 3. Results and Discussion

As material combinations, a Bi_2_Te_3_-based TE module (module 1), a half-Heusler-based TE module (module 2) and an oxide-based TE module (module 3) were chosen. The respective optimized geometries *A*_n_/*A*_p_ for maximum *zT*, matching *I*_q,SC_ and for maximum electrical power are shown in Table 2. For module 1 and 2, all of the optimizations led to very similar *A*_n_/*A*_p_ ratios. Therefore, only the *zT*-optimized modules have been simulated. For module 3, the resulting *A*_n_/*A*_p_ ratios vary widely, so simulations of this module were done for all the calculated optimized geometries.

### 3.1. Similar Material Properties

For the materials that were chosen for module 1 and 2, the optimizations of the *A*_n_/*A*_p_ ratios for maximum *zT*, matching material working points and for maximum power output all result in ratios near 1, with only a slight variation. This is a result of the fairly similar thermoelectric properties of the respective *n*- and *p*-types. Therefore, a fixed *A*_n_/*A*_p_ ratio of 1.04 and 1.08 are used for the simulations of module 1 and module 2, respectively. Note that, although the calculated optimum *A*_n_/*A*_p_ ratios for module 1 and 2 all are close together, they are not the same, meaning that an optimization for maximum power output may still result in a slightly higher power output of the respective module compared to a *zT* optimization. However, the effect is much stronger for the oxide-based module 3, which is the reason why this module is analyzed in depth for all three optimized geometries.

#### 3.1.1. Bi_2_Te_3_-Based TE Module

**Figure 3:**
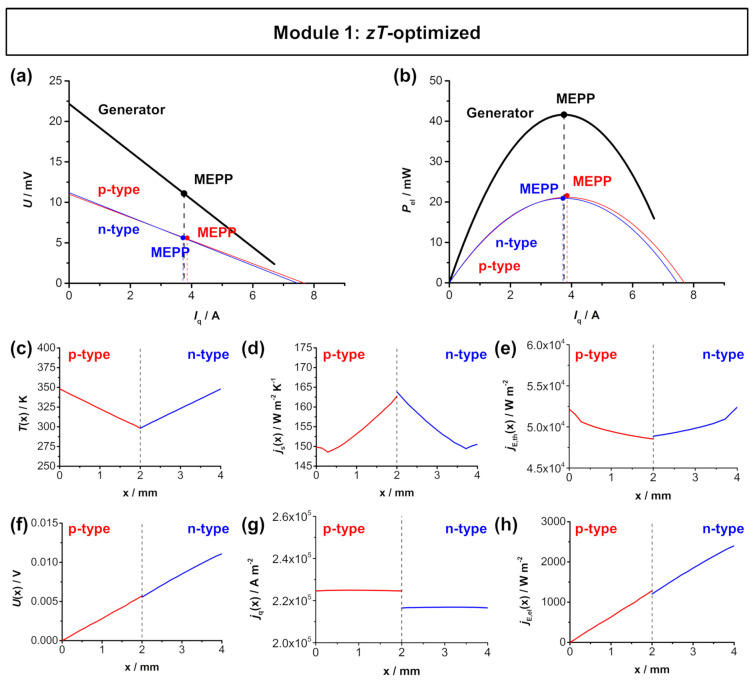
FEM simulations of module 1 (*p*-type Bi_0.5_Sb_1.5_Te_3_ and *n*-type Bi_2_Te_3-x_Sb_x_) with a hot side temperature of 348 K and cold side temperature of 298 K. (**a**) *U*-*I*_q_ characteristics and (**b**) electrical power output *P*_el_–*I*_q_ of the module. The respective MEPPs of the materials overlap and result in a high power output of the TE module. Thermoelectric characteristics of the respective materials as a function of the length of the respective legs: (**c**) temperature *T*(*x*), (**d**) entropy flux density *j*_S_(*x*), (**e**) thermal energy flux density *j*_E,th_(*x*), (**f**) voltage *U*(*x*), (**g**) electrical flux density *j*_q_(*x*), and (**h**) electrical energy flux density *j*_E,el_(*x*) trend throughout one thermocouple. Note that the dashed line in (**c**–**h**) represents the metallic connector between the *p*-type and *n*-type materials. The simulated distributions are shown in Figure A2 in Appendix E.

For module 1, Bi_2_Te_3-x_Sb_x_ [38] and Bi_0.5_Sb_1.5_Te_3_ [37] were chosen as *n*- and *p*-type materials, respectively. As *A*_n_/*A*_p_ ratio, the *zT*-optimized ratio of 1.04 was used in the simulation. Figure 3 shows the simulated *U*-*I*_q_ characteristics and the electrical power output of the Bi_2_Te_3_-based TE module and the respective thermoelectric parameters across the length of the respective legs. The working points of the *p*- and *n*-type material with a *zT*-optimized *A*_n_/*A*_p_ ratio show a good overlap. This results in a high electrical power output of the TE module with a maximum power density ω_el,max,TEG_ of approximately 124.5 mW cm^−2^ at the applied temperature difference of 50 K. The individual fluxes that are within in the *p*-type and *n*-type thermolegs are shown in Figure 3c–h. The temperature is set to be 348 K at the hot side and 298 K at the cold side. The entropy flux density *j*_S_(*x*) and therefore also the thermal energy flux density *j*_E,th_(*x*) are very similar in the respective legs, due to the similar thermal conductivity of the chosen materials. At the applied temperature difference of 50 K, a voltage *U*(*x*) of 11 mV is achieved in one thermocouple. Analogous to the entropy flux density, the electrical flux density *j*_q_(*x*) is also similar in the *p*-type and *n*-type thermolegs. In one thermocouple, this results in an electrical energy flux density *j*_E,el_(*x*) of 2.4 × 10^−3^ W m^−2^. Note that the dashed lines presented in Figure 3c–h represent the metallic connector between the *p*-type and *n*-type materials, so both materials are not in direct contact. Thus the different fluxes do not necessarily have the same value at the dashed line.

#### 3.1.2. Half-Heusler-Based TE Module

**Figure 4:**
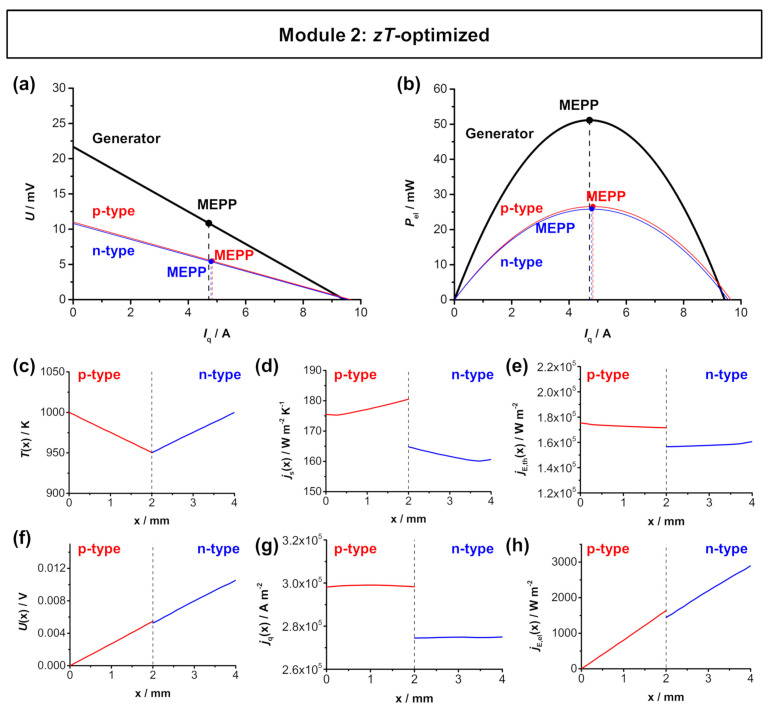
FEM simulations of module 2 (*p*-type FeNb_0.88_Hf_0.12_Sb and *n*-type Hf_0.6_Zr_0.4_NiSn_0.995_Sb_0.005_) with a hot side temperature of 1000 K and cold side temperature of 950 K. (**a**) *U*-*I*_q_ characteristics and (**b**) electrical power output *P*_el_–*I*_q_ of the module. The respective MEPPs of the materials overlap and result in a high power output of the module. Thermoelectric characteristics of the respective materials as a function of the length of the respective legs: (**c**) temperature *T*(*x*), (**d**) entropy flux density *j*_S_(*x*), (**e**) thermal energy flux density *j*_E,th_(*x*), (**f**) voltage *U*(*x*), (**g**) electrical flux density *j*_q_(*x*), and (**h**) electrical energy flux density *j*_E,el_(*x*) trend throughout one thermocouple. Note that the dashed line in (**c**–**h**) represent the metallic connector between the *p*-type and *n*-type materials. The simulated distributions are shown in Figure A3 in Appendix E.

Figure 4 shows the simulated *U*-*I*_q_ characteristics and the electrical power output of the half-Heusler-based TE module and the respective thermoelectric parameters across the length of the respective legs. For *n*- and *p*-type materials, Hf_0.6_Zr_0.4_NiSn_0.995_Sb_0.005_ [40] and FeNb_0.88_Hf_0.12_Sb [39] were chosen.

Analogous to the Bi_2_Te_3_-based module, the materials exhibit similar thermoelectric properties and the resulting *A*_n_/*A*_p_ ratio is still near 1. For the simulations, the *zT*-optimized *A*_n_/*A*_p_ ratio of 1.08 was used. The material working points also show a good overlap as a result of the *zT* optimization. Therefore, the module’s MEPP and MCEP are also close together. The TE module reaches a high electrical power output of approximately 51.1 mW. With an effective area of 0.334 cm^2^, this corresponds to a similarly high maximum power density ω_el,max,TEG_ of 153.14 mW cm^−2^, which is slightly higher compared to the Bi_2_Te_3_-based module 1. The individual fluxes within in the *p*-type and *n*-type thermolegs are shown in Figure 4c–h. The temperature is set to be 1000 K at the hot side and 950 K at the cold side. At the applied 50 K temperature difference, a voltage *U*(*x*) of 10.54 mV can be reached, which is slightly lower compared to the Bi_2_Te_3_-based module, as a result of the slightly lower Seebeck coefficient of the *n*-type material. The entropy flux density *j*_S_(*x*) of the *p*-type is slightly higher when compared to the *n*-type thermoleg, due to the higher thermal conductivity of the *p*-type material. Analogously, the electrical flux density *j*_q_(*x*) is also slightly higher in the *p*-type material, due to the higher electrical conductivity of the *p*-type material. In one thermocouple, a thermal energy flux density *j*_E,th_(*x*) of 16 × 10^3^ W m^−2^ and an electrical energy flux density *j*_E,el_(*x*) of 2.9 × 10^3^ W m^−2^ are reached, both being higher when compared to the Bi_2_Te_3_-based module, due to the higher values of electrical and thermal conductivity of the respective materials.

Table 3 summarizes the simulated characteristics of the Bi_2_Te_3_-based and half-Heusler-based TE modules. The respective material working points are close together, which results in a high electrical power output and conversion efficiency of both modules. However, the Bi_2_Te_3_-based module 1 reaches a higher conversion efficiency of 2.5%, while the half-Heusler based module 2 reaches a higher power output of up to 153 mW cm^−2^. This is the expected behavior, due to the higher power factor, but simultaneously higher thermal conductivity of the half-Heusler materials. This also displays the aforementioned importance of the power factor (for power output), which, for certain applications, may be equally important as the figure of merit *zT* (for efficiency).

### 3.2. Dissimilar Material Properties

For *n*- and *p*-type materials of module 3, In_1.995_Sn_0.05_O_3_ and Ca_3_Co_4_O_9_ [41] were chosen. For these materials, the optimizations of the *A*_n_/*A*_p_ ratios for maximum *zT*, matching *I*_q,SC_ and for maximum power output result in dissimilar ratios of 0.06, 0.13, and 0.24, respectively. Therefore, modules with all calculated *A*_n_/*A*_p_ ratios were simulated.

#### Oxide-Based TE Module

**Figure 5:**
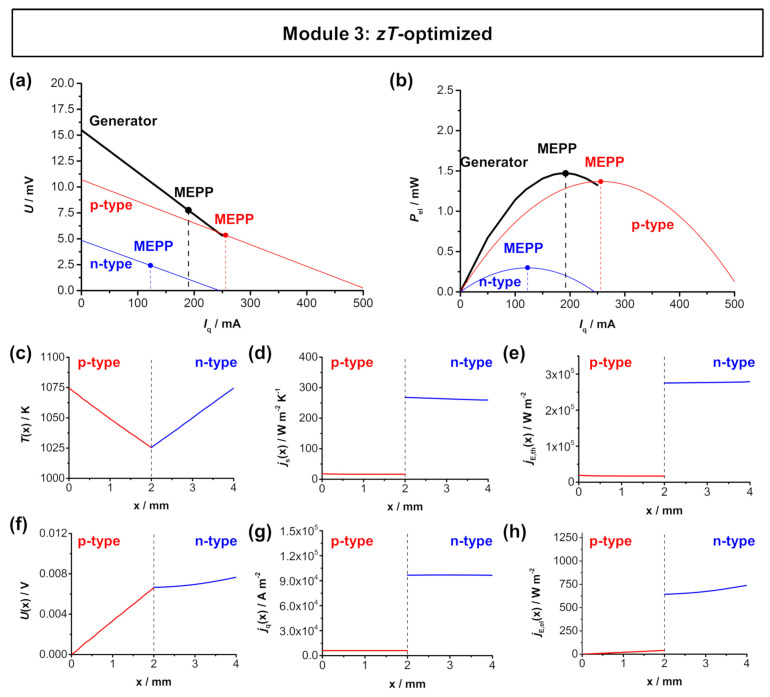
FEM simulations of the *zT*-optimized module 3 (*p*-type Ca_3_Co_4_O_9_ and *n*-type In_1.95_Sn_0.05_O_3_) with a hot side temperature of 1050 K and cold side temperature of 1000 K. (**a**) *U*-*I*_q_ characteristics and (**b**) electrical power output *P*_el_-*I*_q_ of the module. Thermoelectric characteristics of the respective materials as a function of the length of the respective legs: (**c**) temperature *T*(*x*), (**d**) entropy flux density *j*_S_(*x*), (**e**) thermal energy flux density *j*_E,th_(*x*), (**f**) voltage *U*(*x*), (**g**) electrical flux density *j*_q_(*x*), and (**h**) electrical energy flux density *j*_E,el_(*x*) trend throughout one thermocouple. Note, that the dashed line in (**c**–**h**) represent the metallic connector between the *p*-type and *n*-type materials. The simulated distributions are shown in Figure A4 in Appendix E.

Figure 5 shows the simulated *U*-*I*_q_ characteristics and the electrical power output of the Ca_3_Co_4_O_9_-In_1.95_Sn_0.05_O_2_ TE module and the thermoelectric parameters across the length of the respective legs. Here, the *zT* optimization of the *A*_n_/*A*_p_ ratio does not result in an overlap of the respective material working points. The short-circuited electrical current *I*_q,SC_ of the *p*-type Ca_3_Co_4_O_9_ is approximately twice the short-circuited current *I*_q,SC_ of the *n*-type In_1.95_Sn_0.05_O_2_. Therefore, the resulting MEPP of the TE module is located between the respective material working points, and the power output of the module is only slightly higher when compared to the power output of the *p*-type Ca_3_Co_4_O_9_ leg. With an effective area of 0.3332 cm^2^ the simulated TE module reaches a maximum electrical power density ω_el,max,TEG_ of approximately 4.5 mW cm^−2^. The individual fluxes within in the *p*-type and *n*-type thermolegs are shown in Figure 4c–h. The temperature difference was again set to 50 K, with a hot side temperature of 1075 K and a cold side temperature of 1025 K. The strong difference of the Seebeck coefficient of *n*- and *p*-type materials is displayed in the distribution of the voltage *U*(*x*). In the *p*-type material, a voltage of 6.6 mV is reached, while, in the *n*-type material, the voltage only increases by 1 mV to 7.6 mV. The strong difference of thermoelectric properties of *p*- and *n*-type materials is also displayed in the flux density of charge and flux density of entropy, both being higher in the *n*-type In_1.995_Sn_0.05_O_3_ due to the higher electrical and thermal conductivity. Therefore, the same behavior is noticeable in the flux densities of thermal energy and electrical energy.

**Figure 6:**
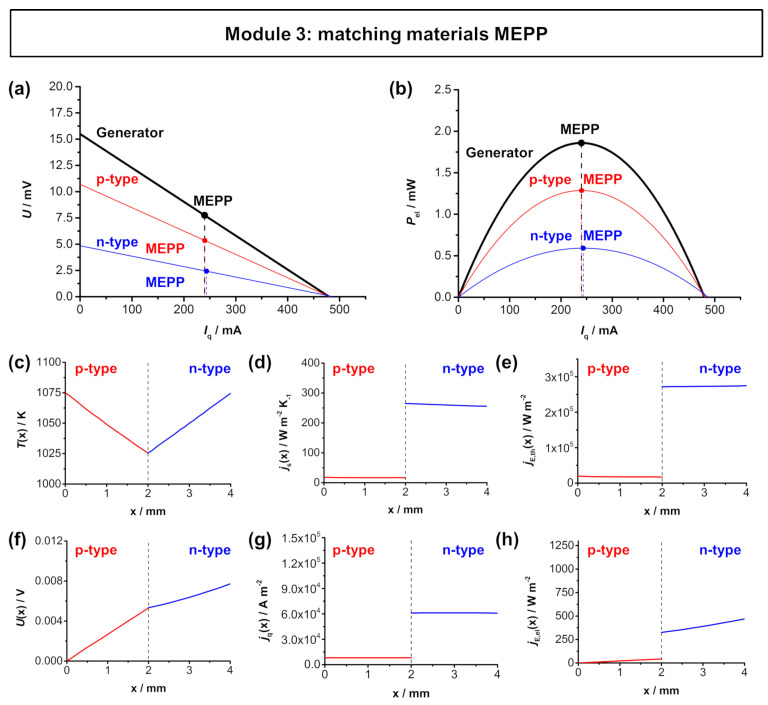
FEM simulations of the module 3 (*p*-type Ca_3_Co_4_O_9_ and *n*-type In_1.95_Sn_0.05_O_3_) with matching *I*_q,SC_ with a hot side temperature of 1050 K and cold side temperature of 1000 K. (**a**) *U*–*I*_q_ characteristics and (**b**) electrical power output *P*_el_-*I*_q_ of the module. Thermoelectric characteristics of the respective materials as a function of the length of the respective legs: (**c**) temperature *T*(*x*), (**d**) entropy flux density *j*_S_(*x*), (**e**) thermal energy flux density *j*_E,th_(*x*), (**f**) voltage *U*(*x*), (**g**) electrical flux density *j*_q_(*x*), and (**h**) electrical energy flux density *j*_E,el_(*x*) trend throughout one thermocouple. Note, that the dashed line in (**c**–**h**) represent the metallic connector between the *p*-type and *n*-type materials. The simulated distributions are shown in Figure A5 in Appendix E.

Figure 6 shows the simulated *U*-*I*_q_ characteristics and the electrical power output of the Ca_3_Co_4_O_9_–In_1.95_Sn_0.05_O_2_ TE module and the respective thermoelectric parameters across the length of the respective legs for an optimized *A*_n_/*A*_p_ ratio for matching *I*_q,SC_. As a result of this optimization, the module MEPP is also similar to the both materials’ working points and the power output of the module is already significantly higher than of the respective materials. With an effective area of 0.333 cm^2^ a maximum electrical power density ω_el,max,TEG_ of approximately 5.64 mW cm^−2^ can be reached. The individual fluxes within in the *p*-type and *n*-type thermolegs for the module optimized for matching *I*_q,SC_ are shown in Figure 6c–h. When compared to the *zT* optimization, the larger area of the *n*-type In_1.995_Sn_0.05_O_3_ results in a bigger impact of the material, displayed in a higher value of voltage reached in the *n*-type material. Additionally, both the entropy flux density (slightly) as well as the electrical flux density (significantly) of the *n*-type material are lower, due to the larger area, which results in the same trend for the flux densities of thermal energy and electrical energy.

**Figure 7:**
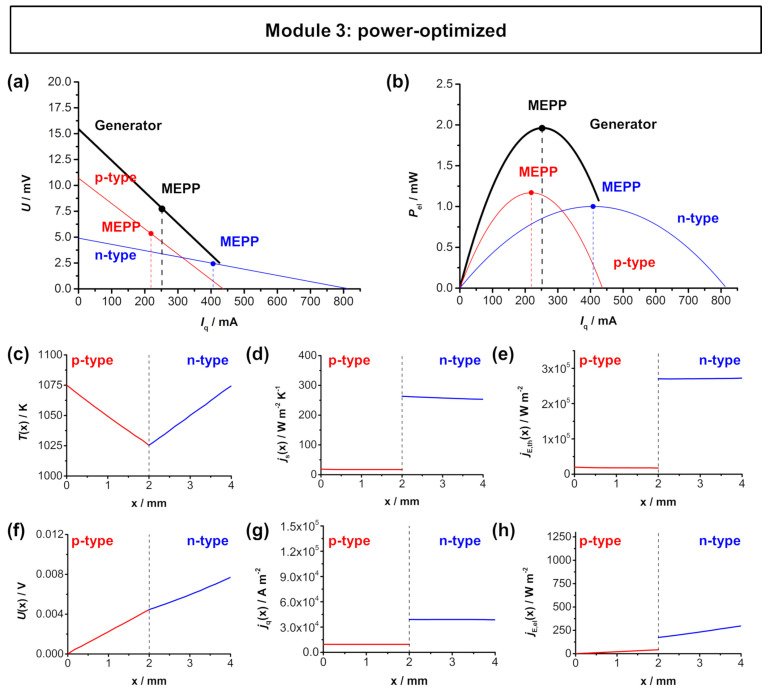
FEM simulations of the power-optimized module 3 (*p*-type Ca_3_Co_4_O_9_ and *n*-type In_1.95_Sn_0.05_O_3_) with a hot side temperature of 1050 K and cold side temperature of 1000 K. (**a**) *U*-*I*_q_ characteristics and (**b**) electrical power output *P*_el_-*I*_q_ of the module. Thermoelectric characteristics of the respective materials as a function of the length of the respective legs: (**c**) temperature *T*(*x*), (**d**) entropy flux density *j*_S_(*x*), (**e**) thermal energy flux density *j*_E,th_(*x*), (**f**) voltage *U*(*x*), (**g**) electrical flux density *j*_q_(*x*), and (**h**) electrical energy flux density *j*_E,el_(*x*) trend throughout one thermocouple. Note, that the dashed line in (**c**–**h**) represent the metallic connector between the *p*-type and *n*-type materials. The simulated distributions are shown in Figure A6 in Appendix E.

Finally, in Figure 7, the power optimization of the *A*_n_/*A*_p_ ratio according to Equation (7) is shown. Again, the material working points do not overlap, but as a result of the increasing cross-sectional area of the *n*-type In_1.95_Sn_0.05_O_2_, the electrical power output of the the *n*-type material is significantly higher compared to the other two optimization strategies. In fact, both materials reach a similar electrical power output *P*_el,max_ of about 1 mW, resulting in a maximum electrical power output *P*_el,max,TEG_ of about 2 mW for the module. This corresponds to a maximum electrical power density ω_el,max,TEG_ of 5.89 mW cm^−2^. The individual fluxes within in the *p*-type and *n*-type thermolegs for the power-optimized module are shown in Figure 7c–h. Here, the trend from the module optimized for matching *I*_q,SC_ continues. The larger area of the *n*-type material results in a higher voltage *U*(*x*) and as well as decreasing flux densities of entropy *j*_S_(*x*) (slightly lower) and charge *j*_q_(*x*) (significantly lower).

Table 4 summarizes the simulated characteristics of the *zT*-optimized Ca_3_Co_4_O_9_-In_1.95_Sn_0.05_O_2_ TE module, the optimized module for matching *I*_q,SC_, as well as for the power-optimized geometry. The module with power-optimized *A*_n_/*A*_p_ ratio reaches a maximum power density of 5.89 mW cm^−2^, which is slightly higher compared to the module with overlapping material working points and about 30% higher when compared to the module with *zT*-optimized geometry. Additionally, the maximum first-law energy conversion efficiency η_I,TEG,max_ for all three optimized geometries have been calculated. As expected, the *zT*-optimized module reaches the highest η_I,TEG,max_ with 0.13%, while the module optimized for matching *I*_q,SC_ and the power-optimized module show slightly lower efficiencies of 0.11% and 0.09%, respectively. This shows the contrary trend of a higher efficiency (for the *zT*-optimized module) and of higher power density (for the power-optimized module).

**Figure 8:**
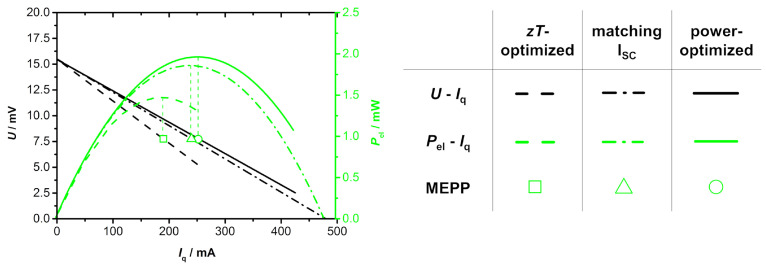
Comparison of all three optimization strategies for module 3 (dash: *zT*-optimized, dash-dot: matching *I*_q,SC_, line: power-optimized). The power-optimized module shows a significantly higher power output when compared to *zT*-optimized module. The module with overlapping material working points is in between, but closer to the maximum power output.

As a result, module 3 is build based on the same materials with identical thermoelectric properties, but it is either optimized for maximum *zT*, matching *I*_q,SC_ or maximum power output. Figure 8 summarizes the results of all three optimization strategies. The *zT* optimization leads to a module with the highest conversion efficiency, but the lowest electrical power output. Contrary, the power optimization leads to a module with the the highest electrical power output, but the lowest conversion efficiency. The module with optimized geometry for matching *I*_q,SC_ is in between, but closer to the maximum electrical power output. This also corresponds to the results of Xing et al. [36], who observed a similar increase in the maximum electrical power output with a respective geometry optimization when compared to an optimization for maximum *zT*. Note that this correlation between the deviation of optimum power output and optimum conversion efficiency is here shown on the example of module 3, but also applies for the other TE modules. As shown in Table 2, the optimum *A*_n_/*A*_p_ ratio for the Bi_2_Te_3_-based module 1 and the half-Heusler-based module 2 also varies slightly for the different optimization strategies. Therefore, also for quite similar thermoelectric materials, a slight deviation between optimum power output and energy conversion efficiency can be expected.

## 4. Conclusions

Three different optimization strategies for the *A*_n_/*A*_p_ ratio were applied, whereas, for certain modules, they all resulted in different geometries. For module 3, based on strongly dissimilar thermoelectric properties of the *p*-type Ca_3_Co_4_O_9_ and the *n*-type In_1.95_Sn_0.05_O_3_, the geometry optimizations show strongly dissimilar *A*_n_/*A*_p_ ratios. Here, a strong deviation between high conversion efficiency (with *zT*-optimized geometry) and high power output (with power-optimized geometry) was found. The power optimization resulted in a 30% higher power output compared to the *zT*-optimized counterpart. For modules with more similar thermoelectric properties of the *n*- and *p*-type, which, in this work, are the Bi_2_Te_3_-based module 1 and the half-Heusler-based module 2, the respective optimum geometries only differ slightly, but also show this deviation in the geometry optimization. This emphasizes that, for TE module concepts, various optimization strategies may be applied, either to target high conversion efficiency or high power output. This phenomena correlates to the diversity of the thermoelectric materials that were used for the TE module. Additionally, this also underlines the similar importance of the power factor of thermoelectric materials, to target a high power output, when compared to the figure of merit *zT*.

## Figures and Tables

**Table 1:** Material combinations for the simulated modules with according literature for the thermoelectric properties. The exact input values are shown in Table A1, Table A2 and Table A3 in Appendix A. For all modules, a stable temperature difference of 50 K has been assumed. For the calculation of the *A*_n_/*A*_p_ ratios, a linear behavior has been assumed and the calculation was done with the medium values of the respective temperature range.

Module	*p*-Type	*n*-Type	*T*_hot_/K	*T*_cold_/K
Module 1	Bi_0.5_Sb_1.5_Te_3_ [37]	Bi_2_Te_3-x_Sb_x_ [38]	348	298
Module 2	FeNb_0.88_Hf_0.12_Sb [39]	Hf_0.6_Zr_0.4_NiSn_0.995_Sb_0.005_ [40]	1000	950
Module 3	Ca_3_Co_4_O_9_ [41]	In_1.95_Sn_0.05_O_3_ [41]	1075	1025

**Table 2:** Resulting optimized geometries according to the *zT* optimization, matching *I*_q,SC_ and power optimization. For the values in brackets, no simulations were carried out, due to insignificant deviation from the *zT* optimization.

Module	[$\frac{An}{Ap}$]_*zT*_	[$\frac{An}{Ap}$]_matching *I*_q,SC__	[$\frac{An}{Ap}$]_power_
Module 1	1.0345	(1.0745)	(1.0459)
Module 2	1.0831	(1.0969)	(1.0308)
Module 3	0.0596	0.1306	0.2433

**Table 3:** Resulting maximum electrical power output *P*_el,max,TEG_, electrical power density ω_el,max,TEG_ and maximum first-law energy conversion efficiency η_I,TEG,max_ of module 1 (Bi_2_Te_3_) and module 2 (half-Heusler materials) for *zT*-optimized geometry.

Module	Module MEPP/A	P_el,max,TEG_/mW	ω_el,max,TEG_/mW cm^−2^	η_I,TEG,max_
Module 1	3.75	41.60	124.50	2.50
Module 2	4.72	51.10	153.14	0.97

**Table 4:** Resulting maximum electrical power output *P*_el,max,TEG_, electrical power density ω_el,max,TEG_, and maximum first-law energy conversion efficiency η_I,TEG,max_ of module 3 with optimized geometry for maximum *zT*, matching *I*_q,SC_ and maximum power output. The resulting power density increases due to the overlapping material working points.

Module	Module MEPP/mA	*P*_el,max,TEG_/mW	ω_el,max,TEG_/mW cm^−2^	η_I,TEG,max_
*zT*-optimized	189.90	1.50	4.51	0.13%
same *I*_q,SC_	239.89	1.86	5.64	0.11%
power-optimized	252.00	1.96	5.89	0.09%

## Abbreviations

The following abbreviations are used in this manuscript:

TE module	thermoelectric module
TEG	thermoelectric generator
MCEP	maximum conversion efficiency point
MEPP	maximum electrical power point
OC	(electrical) open-circuit
SC	(electrical) short-circuit

## Symbols

The following symbols are used in this manuscript:

*Geometry*

*A*	cross-sectional area of thermoelectric material
*A*_n_	cross-sectional area of *n*-type material
*A*_p_	cross-sectional area of *p*-type material
*l*	length of thermoelectric material
*l*_n_	length of *n*-type material
*l*_p_	length of *p*-type material
$\frac{An}{Ap}$	ratio of the cross-sectional areas of the *n*-type and *p*-type materials
$[\frac{An}{Ap}]\mathrm{zT}$	ratio of the cross-sectional areas of the *n*-type and *p*-type materials for maximum *zT*
$[\frac{An}{Ap}]\mathrm{matching}I_{q,\mathrm{SC}}$	ratio of the cross-sectional areas of the *n*-type and *p*-type materials for matching *I*_q,SC_
${\left[\frac{An}{Ap}\right]}_{\mathrm{power}}$	ratio of the cross-sectional areas of the *n*-type and *p*-type materials for maximum power

*Material properties*

α	Seebeck coefficient
α_n_	Seebeck coefficient of *n*-type material
α_p_	Seebeck coefficient of *p*-type material
λ_n_	heat conductivity of *n*-type material
λ_p_	heat conductivity of *p*-type material
λ_OC_	heat conductivity under electrically open-circuited (OC) conditions
Λ_OC_	entropy conductivity under electrically open-circuited (OC) conditions
ρ	specific electrical resistivity
ρ_n_	specific electrical resistivity of *n*-type material
ρ_p_	specific electrical resistivity of *p*-type material
*R*_n_	resistance of *n*-type material
*R*_p_	resistance of *p*-type material
σ	isothermal electrical conductivity
*f*	figure of merit (as introduced by Zener [43]
zT	figure of merit (as introduced by Ioffe [10])

*Thermodynamic potentials*

φ	electric potential
*T*	absolute temperature
*T*_cold_	temperature of the thermoelectric material at its cold side
*T*_hot_	temperature of the thermoelectric material at its hot side
∇T	gradient of the temperature
ΔT	difference of temperature (along the thermoelectric material)
*U*	voltage
*U*_OC_	voltage at electrically open-circuited (OC) conditions

*Fluxes*

*i*	normalized electrical current
*I*_q_	electrical current
*I*_q,SC_	electrical current at electrically short-circuited (SC) conditions
j_q_	electrical flux density
j_s_	entropy flux density
j_E,el_	electrical energy flux density
j_E,th_	thermal energy flux density
*q*	electric charge
*S*	entropy

*Performance*

*P*_el,max_	maximum electrical power output of the thermoelectric material (at MEPP)
*P*_el,max,TEG_	maximum electrical power output of the module (at MEPP)
ω_el,max,TEG_	maximum electrical power density of the module (at MEPP)
*I*_q,MEPP_	current *I*_q_ at the MEPP
*I*_q,MEPP,n_	current *I*_q_ at the MEPP of the *n*-type material
*I*_q,MEPP,p_	current *I*_q_ at the MEPP of the *p*-type material
*U*_MEPP,TEG_	voltage *U* at the MEPP of the TE module
*I*_q,MEPP,TEG_	current *I*_q_ at the MEPP of the TE module
*R*_TEG_	internal resistance of the TE module
η_II,mat_	second-law energy conversion efficiency of a thermoelectric material
η_I,TEG,max_	maximum first-law energy conversion efficiency of the TE module
η_Carnot_	Carnot efficiency of the TE module
η_II,TEG,max_	maximum second-law energy conversion efficiency of the TE module

## Appendix A. Input Data for FEM-Simulation

**Table A1:** Thermoelectric parameters of module 1 (*p*-type Bi_0.5_Sb_1.5_Te_3_ [37], *n*-type Bi_2_Te_3-x_Sb_x_ [38]) used for FEM simulations. For all material parameters a linear behavior within the applied temperature range has been assumed and the average value has been used for the calculation of the *A*_n_/*A*_p_ ratio.

	*p*-Type		*n*-Type	
T/K	348	298	348	298
σ/S cm^−1^	760	990	711	875
α/μV K^−1^	227	213	−228	−220
λ_OC_/W m^−1^ K^−1^	1.31	1.39	1.40	1.35
Λ_OC_/W m^−1^ K^−2^	3.76 × 10^−3^	4.66 × 10^−3^	4.02 × 10^−3^	4.53 × 10^−3^

**Table A2:** Thermoelectric parameters of module 2 (*p*-type FeNb_0.88_Hf_0.12_Sb [39], *n*-type Hf_0.6_Zr_0.4_NiSn_0.995_Sb_0.005_ [40]) used for FEM simulations. For all material parameters a linear behavior within the applied temperature range has been assumed and the average value has been used for the calculation of the *A*_n_/*A*_p_ ratio.

	*p*-Type		*n*-Type	
T/K	1000	950	1000	950
σ/S cm^−1^	1053	1158	960	1000
α/μV K^−1^	223	217	−212	−219
λ_OC_/W m^−1^ K^−1^	4.33	4.44	4.16	3.90
Λ_OC_/W m^−1^ K^−2^	4.33 × 10^−3^	4.76 × 10^−3^	4.16 × 10^−3^	4.11 × 10^−3^

**Table A3:** Thermoelectric parameters of module 3 (*p*-type Ca_3_Co_4_O_9_ [41], *n*-type In_1.95_Sn_0.05_O_3_ [41]) used for FEM simulations. For all material parameters a linear behavior within the applied temperature range has been assumed and the average value has been used for the calculation of the *A*_n_/*A*_p_ ratio.

	*p*-Type		*n*-Type	
T/K	1075	1025	1075	1025
σ/S cm^−1^	29.63	31.48	609.26	448.15
α/μV K^−1^	202.83	225.74	−100.94	−92.45
λ_OC_/W m^−1^ K^−1^	0.63	0.66	10.70	10.94
Λ_OC_/W m^−1^ K^−2^	0.59 × 10^−3^	0.64 × 10^−3^	9.95 × 10^−3^	10.67 × 10^−3^

## Appendix B. A_n_/A_p_ Optimization for Matching Short-Circuit Current

Here, the optimized *A*_n_/*A*_p_ ratio for matching *I*_q,SC_ is derived. The idea of this optimization is as follows: the working points of the respective thermoelectric materials overlap, if the flux of charge in both materials is the same. Then, the working points of the materials overlap, so

(A1) $$I_{q,\mathrm{MEPP},n}=I_{q,\mathrm{MEPP},p}$$

By including

(A2) $$I_{q,\mathrm{MEPP},n}=\frac{|\alpha_{n}{|\left(\Delta T\right)}^{2}}{2R_{n}}$$

and

(A3) $$I_{q,\mathrm{MEPP},p}=\frac{\alpha_{p}{\left(\Delta T\right)}^{2}}{2R_{p}}$$

with the electrical resistance of the materials

(A4) $$R_{n}=\rho_{n}\cdot \frac{l_{n}}{A_{n}}$$

and

(A5) $$R_{p}=\rho_{p}\cdot \frac{l_{p}}{A_{p}}$$

the following relation is received:

(A6) $$\frac{|\alpha_{n}{|\cdot \left(\Delta T\right)}^{2}}{2\rho_{n}\frac{l_{n}}{A_{n}}}=\frac{\alpha_{p}\cdot {\left(\Delta T\right)}^{2}}{2\rho_{p}\frac{l_{p}}{A_{p}}}$$

After rearrangement and with the assumed same length of the thermolegs *l*_n_=*l*_p_ the result for a *A*_n_/*A*_p_ ratio for matching *I*_q,SC_ is:

(A7) $$\frac{A_{n}}{A_{p}}=\frac{\alpha_{p}}{|\alpha_{n}|}\cdot \frac{\rho_{n}}{\rho_{p}}$$

## Appendix C. A_n_/A_p_ Optimization for Maximum Power

The maximum power output of a TE module is a function of the electrical current *I*_q_ of the module at the MEPP *I*_q,MEPP,TEG_ and the voltage *U* of the module at the MEPP *U*_MEPP,TEG_, which are calculated according to Equations (A8) and (A9):

(A8) $$I_{q,\mathrm{MEPP},\mathrm{TEG}}=\frac{(\alpha_{p}-\alpha_{n})\cdot \Delta T}{2R}$$

(A9) $$U_{\mathrm{MEPP},\mathrm{TEG}}=\frac{(\alpha_{p}-\alpha_{n})\cdot \Delta T}{2}$$

From this, the maximum electrical power output of a module at the MEPP can be derived as

(A10) $$P_{\mathrm{el},\max,\mathrm{TEG}}=\frac{{\left(\alpha_{p}-\alpha_{n}\right)}^{2}\cdot {\left(\Delta T\right)}^{2}}{4R_{\mathrm{TEG}}}$$

with the internal electrical resistance of the module *R*_TEG_

(A11) $$R_{\mathrm{TEG}}=\rho_{p}\frac{l_{p}}{A_{p}}+\rho_{n}\frac{l_{n}}{A_{n}}$$

Considering, that the effecive area *A* is a sum of the cross-sectional areas *A*_n_ and *A*_p_, Equation (A11) can be differentiated and has to be equal 0 for its maximum. So

(A12) $$-{\left(\alpha_{p}-\alpha_{n}\right)}^{2}{\left(\Delta T\right)}^{2}\cdot \frac{\frac{\rho_{p}l_{p}}{{\left(A+A_{p}\right)}^{2}}-\frac{\rho_{n}l_{n}}{A_{p}^{2}}}{{\left(\frac{\rho_{p}l_{p}}{A_{p}}+\frac{\rho_{n}l_{n}}{A-A_{p}}\right)}^{2}}=0$$

This Equation (A12) is zero, if the numerator of the fraction is zero, so

(A13) $$\frac{\rho_{p}l_{p}}{{\left(A+A_{p}\right)}^{2}}-\frac{\rho_{n}l_{n}}{A_{p}^{2}}=0$$

After rearrangement, the optimum *A*_n_/*A*_p_ ratio for maximum power output is received as

(A14) $$\frac{A_{n}}{A_{p}}=\sqrt{\frac{\rho_{n}}{\rho_{p}}}$$

The final Equation (A14) derived corresponds to the reported ratio for maximum power output of Xing et al. [36].

## Appendix D. Efficiency of the Module

The maximum first-law efficiency η_I,TEG,max_ of a module is the product of the Carnot efficiency η_Carnot_ and the second-law efficiency η_II,TEG,max_ [9,11] and can be determined as

(A15) $$\eta_{I,\mathrm{TEG},\max}=\eta_{\mathrm{Carnot}}\cdot \eta_{\mathrm{II},\mathrm{TEG},\max}=\frac{T_{\mathrm{hot}}-T_{\mathrm{cold}}}{T_{\mathrm{hot}}}\cdot \frac{\sqrt{1+Z\bar{T}}-1}{\sqrt{1+Z\bar{T}}+1}$$

Here, $\bar{T}$ is the average temperature and *Z* is a function of the materials thermoelectric parameters:

(A16) $$Z=\frac{\alpha^{2}}{R\cdot K}$$

with

(A17) $$\alpha =(\alpha_{p}-\alpha_{n})$$

(A18) $$R=\frac{1}{\sigma_{p}}\cdot \frac{l_{p}}{A_{p}}+\frac{1}{\sigma_{n}}\cdot \frac{l_{n}}{A_{n}}$$

and

(A19) $$K=\lambda_{p}\cdot \frac{A_{p}}{l_{p}}+\lambda_{n}\cdot \frac{A_{n}}{l_{n}}$$

So, the maximum first-law efficiency η_I,TEG,max_ of a module can be determined as a function of the materials thermoelectric parameter and the respective cross-sectional areas *A*_n_ and *A*_p_ [30].

## References

[1] Kishore R.A., Marin A., Wu C., Kumar A., Priya S. (2019). Energy Harvesting—Materials, Physics, and System Design with Practical Examples.

[2] Wolf M., Hinterding R., Feldhoff A. (2019). High Power Factor vs. High zT—A Review of Thermoelectric Materials for High-Temperature Application. Entropy.

[3] Gayner C., Kar K.K. (2016). Recent Advances in Thermoelectric Materials. Prog. Mater. Sci..

[4] He R., Schierning G., Nielsch K. (2018). Thermoelectric Devices: A Review of Devices, Architectures, and Contact Optimization. Adv. Mater. Technol..

[5] Liu X., Wang Z. (2019). Printable Thermoelectric Materials and Applications. Front. Mater..

[6] He W., Zhang G., Zhang X., Ji J., Li G., Zhao X. (2015). Recent Development and Application of Thermoelectric Generator and Cooler. Appl. Energy.

[7] Fuchs H.U. (2014). A Direct Entropic Approach to Uniform and Spatially Continuous Dynamical Models of Thermoelectric Devices. Energy Harvest. Syst..

[8] Feldhoff A. (2015). Thermoelectric Material Tensor Derived from the Onsager-de Groot-Callen Model. Energy Harvest. Syst..

[9] Fuchs H. (2010). The Dynamics of Heat—A Unified Approach to Thermodynamics and Heat Transfer.

[10] Ioffe A.F. (1957). Semiconductor Thermoelements, and Thermoelectric Cooling.

[11] Feldhoff A. (2020). Power Conversion and its Efficiency in Thermoelectric Materials. Entropy.

[12] Mamur H., Bhuiyan M.R., Korkmaz F., Nil M. (2018). A Review on Bismuth Telluride (Bi_2_Te_3_) Nanostructure for Thermoelectric Applications. Renew. Sustain. Energy Rev..

[13] Guo W., Ma J., Zheng W. (2016). Bi_2_Te_3_ Nanoflowers Assembled of Defective Nanosheets with Enhanced Thermoelectric Performance. J. Alloy. Compd..

[14] Zhang J., Wu D., He D., Feng D., Yin M., Qin X., He J. (2017). Extraordinary Thermoelectric Performance Realized in n-Type PbTe through Multiphase Nanostructure Engineering. Adv. Mater..

[15] Zhao L.D., Lo S.H., Zhang Y., Sun H., Tan G., Uher C., Wolverton C., Dravid V.P., Kanatzidis M.G. (2014). Ultralow Thermal Conductivity and High Thermoelectric Figure of Merit in SnSe Crystals. Nature.

[16] Peng K., Lu X., Zhan H., Hui S., Tang X., Wang G., Dai J., Uher C., Wang G., Zhou X. (2016). Broad Temperature Plateau for High zTs in Heavily Doped p-Type SnSe Single Crystals. Energy Environ. Sci..

[17] Shuai J., Mao J., Song S., Zhang Q., Chen G., Ren Z. (2017). Recent Progress and Future Challenges on Thermoelectric Zintl Materials. Mater. Today Phys..

[18] Sun J., Singh D.J. (2017). Thermoelectric Properties of AMg_2_X_2_, AZn_2_Sb_2_ (A = Ca, Sr, Ba; X = Sb, Bi), and Ba_2_ZnX_2_ (X = Sb, Bi) Zintl Compounds. J. Mater. Chem. A.

[19] Chen X., Wu H., Cui J., Xiao Y., Zhang Y., He J., Chen Y., Cao J., Cai W., Pennycook S.J. (2018). Extraordinary Thermoelectric Performance in n-Type Manganese Doped Mg_3_Sb_2_ Zintl: High Band Degeneracy, Tuned Carrier Scattering Mechanism and Hierarchical Microstructure. Nano Energy.

[20] Zhu H., He R., Mao J., Zhu Q., Li C., Sun J., Ren W., Wang Y., Liu Z., Tang Z. (2018). Discovery of ZrCoBi Based Half Heuslers with High Thermoelectric Conversion Efficiency. Nat. Commun..

[21] Poon S.J. (2019). Half-Heusler Compounds: Promising Materials For Mid-To-High Temperature Thermoelectric Conversion. J. Phys. D Appl. Phys..

[22] Yin Y., Tudu B., Tiwari A. (2017). Recent Advances in Oxide Thermoelectric Materials and Modules. Vacuum.

[23] Zhang X., Chang C., Zhou Y., Zhao L.D. (2017). BiCuSeO Thermoelectrics: An Update on Recent Progress and Perspective. Materials.

[24] Cowen L.M., Atoyo J., Carnie M.J., Baran D., Schroeder B.C. (2017). Review—Organic Materials for Thermoelectric Energy Generation. ECS J. Solid State Sci. Technol..

[25] Boudouris B.W., Yee S. (2017). Structure, Properties and Applications of Thermoelectric Polymers. J. Appl. Polym. Sci..

[26] Ashalley E., Chen H., Tong X., Li H., Wang Z.M. (2015). Bismuth Telluride Nanostructures: Preparation, Thermoelectric Properties and Topological Insulating Effect. Front. Mater. Sci..

[27] Gharsallah M., Serrano-Sánchez F., Bermúdez J., Nemes N.M., Martínez J.L., Elhalouani F., Alonso J.A. (2016). Nanostructured Bi_2_Te_3_ Prepared by a Straightforward Arc-Melting Method. Nanoscale Res. Lett..

[28] Kim K., Kim G., Lee H., Lee K.H., Lee W. (2018). Band Engineering and Tuning Thermoelectric Transport Properties of p-type Bi_0.52_Sb_1.48_Te_3_ by Pb Doping for Low-Temperature Power Generation. Scr. Mater..

[29] Ming T., Wu Y., Peng C., Tao Y. (2015). Thermal Analysis on a Segmented Thermoelectric Generator. Energy.

[30] Ouyang Z., Li D. (2016). Modelling of Segmented High-Performance Thermoelectric Generators with Effects of Thermal Radiation, Electrical and Thermal Contact Resistances. Sci. Rep..

[31] Korotkov A.S., Loboda V.V., Makarov S.B., Feldhoff A. (2017). Modeling Thermoelectric Generators Using the ANSYS Software Platform: Methodology, Practical Applications, and Prospects. Russ. Microelectron..

[32] Oshima K., Inoue J., Sadakata S., Shiraishi Y., Toshima N. (2017). Hybrid-Type Organic Thermoelectric Materials Containing Nanoparticles as a Carrier Transport Promoter. J. Electron. Mater..

[33] Culebras M., Igual-Muñoz A.M., Rodríguez-Fernández C., Gómez-Gómez M.I., Gómez C., Cantarero A. (2017). Manufacturing Te/PEDOT Films for Thermoelectric Applications. ACS Appl. Mater. Interfaces.

[34] Wolf M., Menekse K., Mundstock A., Hinterding R., Nietschke F., Oeckler O., Feldhoff A. (2019). Low Thermal Conductivity in Thermoelectric Oxide-Based Multiphase Composites. J. Electron. Mater..

[35] Narducci D. (2011). Do we Really Need High Thermoelectric Figures of Merit? A Critical Appraisal to the Power Conversion Efficiency of Thermoelectric Materials. Appl. Phys. Lett..

[36] Xing Z., Liu R., Liao J., Wang C., Zhang Q., Song Q., Xia X., Zhu T., Bai S., Chen L. (2020). A-Device-To-Material Strategy Guiding the “Double-High” Thermoelectric Module. Joule.

[37] Poudel B., Hao Q., Ma Y., Lan Y., Minnich A., Yu B., Yan X., Wang D., Muto A., Vashaee D. (2008). High-Thermoelectric Performance of Nanostructured Bismuth Antimony Telluride Bulk Allys. Science.

[38] Kim H.S., Kikuchi K., Itoh T., Iida T., Taya M. (2014). Design of Segmented Thermoelectric Generator Based on Cost-Effective and Light-Weight Thermoelectric Alloys. Mater. Sci. Eng. B Solid State Mater. Adv. Technol..

[39] Fu C., Bai S., Liu Y., Tang Y., Chen L., Zhao X., Zhu T. (2015). Realizing High Figure of Merit in Heavy-Band p-Type half-Heusler Thermoelectric Materials. Nat. Commun..

[40] Chen L., Gao S., Zeng X., Mehdizadeh Dehkordi A., Tritt T.M., Poon S.J. (2015). Uncovering High Thermoelectric Figure of Merit in (Hf,Zr)NiSn half-Heusler Alloys. Appl. Phys. Lett..

[41] Bittner M., Geppert B., Kanas N., Singh S.P., Wiik K., Feldhoff A. (2016). Oxide-Based Thermoelectric Generator for High-Temperature Application Using p-Type Ca_3_Co_4_O_9_ and n-Type In_1.95_Sn_0.05_O_3_ Legs. Energy Harvest. Syst..

[42] Bergman T.L., Lavine A.S., Incropera F.P., DeWitt D.P. (2011). Fundamentals of Heat and Mass Transfer.

[43] Zener C. (1961). Putting Electrons to Work. Trans. ASM.

